# Comparison of the Efficacy and Safety Endpoints of Five Therapies for Atrial Fibrillation: A Network Meta-Analysis

**DOI:** 10.3389/fcvm.2022.853149

**Published:** 2022-06-03

**Authors:** Tongyu Wang, Tingting Fang, Zeyi Cheng

**Affiliations:** ^1^Department of Cardiovascular Medicine, The Fourth Affiliated Hospital of China Medical University, Shenyang, China; ^2^Department of Cardiology, The Sixth People’s Hospital Affiliated to Shanghai Jiao Tong University, Shanghai, China; ^3^Department of Cardiac Surgery, Ruijin Hospital, Shanghai Jiaotong University School of Medicine, Shanghai, China

**Keywords:** atrial fibrillation, atrioventricular node ablation, stroke, recurrence, radiofrequency ablation

## Abstract

**Introduction:**

Atrial fibrillation (AF) is a prevalent arrhythmia that occurs in 2–4% of adults and poses a threat to human health. Thus, comparison of the efficacy and safety of therapies for AF is warranted. Here, we used network analysis to compare efficacy (arrhythmia recurrence and re-hospitalization) and safety (ischemic cerebral vascular events, all-cause mortality, and cardiovascular mortality) endpoints among five major therapies for AF.

**Methods:**

The PubMed, Cochrane, and Embase databases were searched, and relevant literature was retrieved. Only studies that made comparisons among the therapies of interest and involved patients with AF were included. Pairwise comparisons and frequentist method (SUCRA plot) analyses were conducted.

**Results:**

In total, 62 studies were included in the pooled analysis. In pairwise comparisons, atrioventricular nodal ablation plus permanent pacemaker (AVN + PPM) was associated with a significantly higher risk of atrial arrhythmia recurrence than surgical ablation [odds ratio (OR): 23.82, 95% confidence interval (CI): 1.97–287.59, fixed-effect model; 3.82, 95% CI: 1.01–559.74, random-effects model]. Furthermore, radiofrequency ablation was associated with a significantly lower risk of cardiovascular mortality than medication in pairwise comparison (OR: 0.49, 95% CI: 0.29–0.83, fixed-effect model; OR: 0.49, 95% CI: 0.27–0.9, random-effects model). Frequentist analysis indicated that AVN + PPM had the best performance in reducing the risk of safety and efficacy endpoints.

**Conclusion:**

Non-pharmaceutical therapies showed superior performance to traditional drug therapy in lowering the risk of safety and efficiency endpoint events. AVN + PPM performed best in reducing the risk of safety and efficacy endpoints.

## Introduction

Atrial fibrillation (AF) is a common arrhythmia characterized by non-synchronized atrial electrical activation on electrocardiogram (ECG) (1) and associated with a high incidence of ischemic stroke and heart failure (2, 3). The current estimated prevalence of AF in adults is between 2 and 4%, and a 2.3-fold rise is expected, posing a burden on healthcare systems worldwide (4).

According to the 2020 European Society of Cardiology (ESC) guideline for the diagnosis and management of AF, rhythm control for reducing symptom onset and improving life quality is recommended at the class IA evidence level (4). Furthermore, a recent study proved that rhythm control is also beneficial to patients in terms of preventing stroke, coronary syndromes, heart failure, and cardiovascular death (5). In addition to traditional drug therapies, various instrumental treatments have been developed, facilitating more effective pathways for rhythm control in patients with AF. The SARA study in 2014 proved that catheter radiofrequency ablation (RFA) is more efficient than drug therapy in reducing AF recurrence (5). Furthermore, cryoballoon ablation (CBA), which was invented to achieve better circumferential pulmonary vein isolation than traditional point-by-point-RFA, exhibits a higher procedure success rate than traditional drug therapy (6); however, there was no difference in efficacy endpoint at 1-year follow-up between patients receiving RFA and CBA. There are also techniques other than RFA and CBA. In patients with AF combined with other cardiovascular disorders, such as valvular disease, who require open chest surgery, surgical procedures for ablation of AF can be performed simultaneously (7). Moreover, the ESC guideline for AF management suggests that surgical ablation (SA) of AF can be considered for those patients where drug therapy fails (8). Recent studies have shown that SA is superior to drug therapy in reducing arrhythmia (9). Nevertheless, relative to RFA, the effectiveness of SA in reducing arrhythmia recurrence is inconsistent and patients undergoing SA have a higher risk of safety endpoint events (10).

To date, studies focused on the comparison of the efficacy of instrumental therapy and traditional drug approaches have generated similar results; however, investigations focused on ranking the efficacy and safety of instrumental therapies are scarce. In this study, we used frequency frame-based network meta-analysis (NMA) to compare efficacy and safety endpoints among drug therapy, RFA, CBA, SA, and atrioventricular node ablation plus permanent pacemaker implantation (AVN + PPM). We chose to conduct NMA because most studies comparing different strategies for AF therapy to date had head-to-head designs, while studies making comparisons among multiple arms are scarce; thus, NMA was selected to overcome the effects of co-variants on comparisons. Moreover, AVN + PPM, which is proven to improve high heart rate and exercise tolerance in patients with AF, has rarely been compared with other instrumental therapies (11), and this is the first study focused on comparing efficacy and safety risks among these five therapies.

## Methods

This study was based on established methods (frequentist-based network meta-analysis) and followed the Preferred Reporting Items for Systematic Reviews and Meta-Analyses (PRISMA) statement for reporting systematic reviews and meta-analyses in healthcare interventions.

### Literature Search and Selection

The Cochrane, PubMed, and Embase databases were thoroughly searched using the following keywords:

#1 cryoballoon ablation
#2 radiofrequency ablation
#3 atrioventricular node ablation
#4 surgical ablation
#5 #1 OR #2 OR #3 OR #4
#6 AF
#7 randomized controlled trial
#8 RCT
#9 #7 OR #8
#10 #5 AND #6 AND #9

All search results were stored using Endnote software for further filtration. The inclusion criteria were as follows:

(1) Patients diagnosed with AF according to the 2020 ESC guidelines for atrial fibrillation management (30-s 12-lead ECG tracing or entire ECG showing the manifestations of AF).
(2) Interventions limited to drug therapy, catheter RFA, CBA, SA, and AVN + PPM.
(3) One of the following outcomes was reported: atrial arrhythmia recurrence [AF/atrial flutter (AFL)/tachycardia (AT) with high heart rate], all-cause mortality, cardiovascular mortality, all-cause re-hospitalization, and stroke.
(4) Studies with two, or multiple, arms.
(5) Study type limited to randomized control trial (RCT).

The exclusion criteria were as follows:

(1) Sample size < 50.
(2) Studies not reporting results, single-arm studies, and conference abstracts.
(3) Study data could not be obtained.
(4) Studies focused on ablation strategies.
(5) Unrelated studies.
(6) Reports in languages other than English.
(7) Duplicate articles.
(8) To reduce the possibility of including replicated studies. Studies using the same study cohorts were excluded; when there were two or more reports of studies using the same cohort, the study with the longest follow-up was included.

### Data Extraction

Two researchers independently assessed the eligibility of included studies and the quality of included articles and used the Cochrane handbook to assess the risk of bias in individual studies. The focus of this analysis was efficacy and safety endpoints. Efficacy endpoints included recurrence of atrial arrhythmia (AF/AFL/AT) and all-cause re-hospitalization. Safety endpoints included all-cause mortality, cardiovascular mortality, and cerebral vascular event [transient ischemia attack (TIA)/stroke]. The number of patients in each treatment group and those with event occurrence for each clinical outcome were recorded. Furthermore, only data collected at the end of the longest follow-up period were included. In studies with a blanking period, the number of patients involved in each treatment was recorded as the number of patients after the blanking period. For outcomes with zero events, 0.1 was substituted as the number of patients with events occurring. In those studies where the exact number of patients in which events occurred was not recorded, data were determined by analyzing the Kaplan–Meier curves.

### Evaluation of the Risk of Bias

To assess the risk of bias in the included studies, we adopted the rules for assessing the risk of bias for a study described in the Cochrane Handbook for Systematic Reviews of Interventions, using Review Manager software (Version 5.3) to plot a risk-of-bias graph. To assess publication bias, we used funnel graphs generated using Stata software (Version 13.0).

### Synthesis Methods and Statistics

Studies that met the inclusion criteria and assessed endpoints of interest were included in the following synthesis processes. For data synthesis, frequency-based network analysis was used to obtain estimates of the relative effects of all interventions on efficacy and safety outcomes, by combining direct and indirect evidence using random-effects and fixed-effects models to assess result stability. Odds ratios were used to evaluate relative risk in pairwise comparisons. All calculations were performed in Stata software (Version 13.0). Relationships determined by comparisons are presented as evidence analysis plots. Contribution plots were used to identify the contributions of direct comparisons to combined comparisons. Comparison method (structural or non-structural) was selected by evaluation of the consistency (i.e., the agreement between direct and indirect evidence) of loops, using “ifplot” in the “network” software package. The inconsistency factor (IF) was used to evaluate differences between direct and indirect comparisons. To test the stability and heterogeneity of results in pairwise comparisons, interval plots showing the results of random- and fixed-effect models were drawn. Surface under the cumulative ranking curve (SUCRA) and mean rank values were used to rank treatments for each outcome.

## Results

### Literature Selection

**FIGURE 1:**
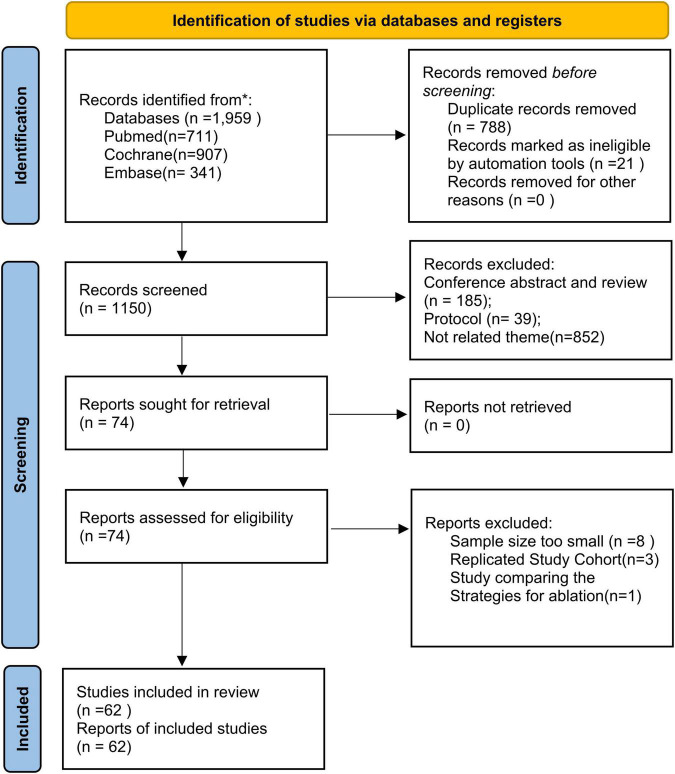
The PRISMA flowchart details the literature selection strategy.

The Embase, PubMed, and Cochrane databases were searched and 1,959 reports, published from 1993 to 2021, were retrieved; however, only 1,938 reports were recorded in the literature management software. First, duplicate publications (*n* = 788) were filtered. Then, 1,076 reports were screened by reading the title and abstract, and 74 records were evaluated by retrieving the full text. Eight studies were excluded because of the limited sample size (*n* < 50). Finally, 62 articles were pooled for subsequent analysis. Figure 1 illustrates the literature screening process. Supplementary Figures 1, 2 illustrate the risk-of-bias assessments.

### Evaluation of Efficacy Endpoints

Atrial arrhythmia recurrence and re-hospitalization rate were selected as endpoints to evaluate the efficacy of the five therapies.

#### Evaluation of Atrial Arrhythmia Recurrence in Patients Undergoing Different Therapies

**FIGURE 2:**
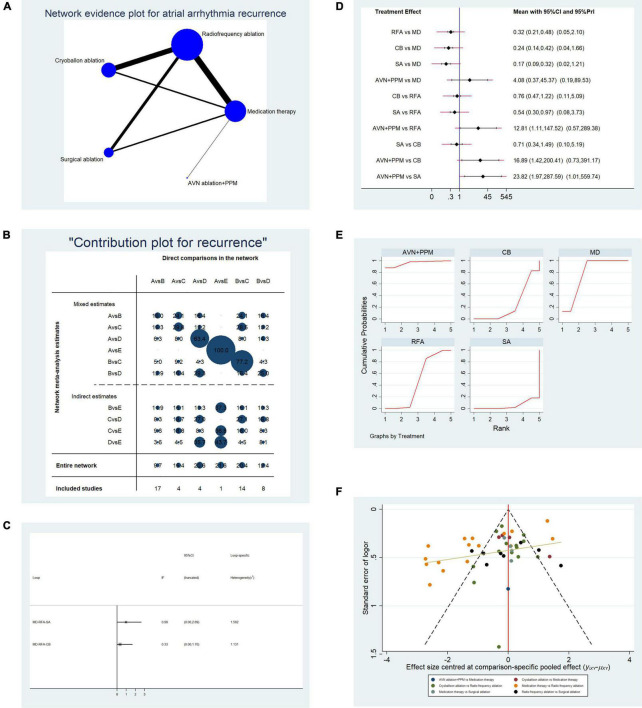
Analysis of risk of atrial arrhythmia in patients undergoing five types of treatment for atrial fibrillation. **(A)** Network evidence plot of atrial arrhythmia (AF/AFL/AT) recurrence risk. Line thickness represents the number of comparisons between the two arms, and node size represents the sample size of each arm. **(B)** Contribution plot shows the contribution of direct comparisons to the combined comparison of atrial arrythmia recurrence risk: A, medication; B, radiofrequency ablation; C, cryoballoon ablation; D, surgical ablation; E, AVN ablation + PPM. **(C)** Ifplot indicates the consistency in each evidence loop. **(D)** Pairwise comparisons of atrial arrhythmia (AF/AFL/AT) recurrence risk among the five therapies for atrial fibrillation. **(E)** SUCRA plots indicate the cumulative possibility of ranking first for the risk of atrial arrhythmia recurrence. **(F)** Funnel plot illustrates the publication bias of included studies.

To assess the impact of different therapies on atrial arrhythmia recurrence rate, data from 48 studies reporting the number of patients with this event were pooled. Basic characteristics of the included studies (age of participants, type of AF, and follow-up duration) are shown in Table 1A. Overall, 9,278 patients were included and atrial arrhythmia recurred in 4,770 patients. Figure 2A presents a network evidence plot; node size represents the number of arms of each treatment, and the thickness of edges indicates the number of direct comparisons. A contribution plot was constructed to illustrate the contribution of each direct comparison to the overall comparison (Figure 2B). Before ranking each treatment, we used a loop-based ifplot to evaluate the consistency of each evidence loop and select the most appropriate model. As shown in Figure 2C, consistency, represented by the difference between direct and indirect comparisons, was acceptable, as the interval of the inconsistency factor (IF) overlapped with the zero-effect line in the two evidence loops. Thus, we selected the structural model for further analysis. Paired comparisons among the five treatments revealed that, relative to traditional medication therapy, RFA [odds ratio (OR): 0.32, 95% confidence interval (CI): 0.21–0.48], CBA (OR: 0.24, 95% CI: 0.14–0.42), and SA (OR: 0.17, 95% CI: 0.09–0.32) were associated with lower risk of atrial arrhythmia recurrence in the fixed-effect model, while in the random-effects model, risk of atrial arrhythmia recurrence did not differ significantly following RFA, SA, and CBA relative to medication. As shown in Figure 2D, the 95% CI values of the random-effects models for CBA, SA, and RFA overlapped the zero-effect line; however, comparison between AVN + PPM strategy and medication therapy for the risk of atrial arrhythmia recurrence showed a stable result, with OR: 4.08, 95% CI: 0.37–45.37 in the fixed-effect model and OR: 4.08, 95% CI: 0.19–89.53 in the random-effects model.

**TABLE 1A:** Basic characteristics of studies included in the analysis of the risk of atrial arrhythmia recurrence.

Author(year)	Arm1						Arm2						design	Type of AF	Follow-UP time
	Number of events	Total number	Age	LVEF%	CAD N(percentage)	Hypertension	Number of events	Total number	age	LVEF%	CAD N(percentage)	Hypertension			
Brignole et al. (11)	2	52	72 ± 9	40 ± 12	19 (36)	39 (75)	1	50	71 ± 12	41 ± 12	13 (26)	35 (70)	ANJPM-MD	Permanent	16 months
Brignole et al. (57)	4	34	72 ± 9	NA	14	NA	2	32	72 ± 9	NA	11	NA	ANJPM-MD	Persistent	12-month
Brignole et al. (58)	12	70	74 ± 9	NA	25 (36)	52 (74)	5	63	72 ± 11	NA	16 (25)	46 (73)	ANJPM-MD	Permanent	2 years
Marrouche et al. (16)	41	184	64	31.5	NA	NA	20	179	64	32.5	NA	NA	RFC-MD	Paroxysmal or persistent	37.8 months
Kuck et al. (21)	6	100	65 ± 8	24.8 ± 8.8	NA	55 (76)	3	98	65 ± 8	27.8 ± 9.5	NA	56 (82)	RFC-MD	Persistent	12 months
Andrade et al. (29)	0	150	58.6 ± 9.2	59.1 ± 6.6	7 (4.7)	55 (36.9)	0	155	58.9 ± 10.3	59.3 ± 6.8	12 (7.8)	57 (37.0)	CB-MD	Paroxysmal	12 months
Wazni et al. (30)	0	100	61.6 ± 11.2	61.1 ± 5.9	12 (12)	57 (58)	0	105	60.4 ± 11.2	60.9 ± 6.0	13 (12)	58 (56)	CB-MD	Paroxysmal	12 months
Andrade et al. (7)	0	116	58.6 ± 9.2	59.1 ± 6.6	6 (5.2)	40 (34.8)	0	232	58.9 ± 10.3	59.3 ± 6.8	19(8.2)	80(35)	CB-RFC	Paroxysmal	12 months
Osmancik et al. (38)	32	99	71.3 ± 7.9	49.6 ± 12.5	65 (65.7)	78 (78.8)	27	108	69.8 ± 7.9	52.2 ± 11.2	55 (50.9)	87 (80.6)	SA-MD	Paroxysmal, persistent, or long-standing persistent	5 years
DeLurgio et al. (39)	0	52	65.1 ± 6.7	55.7 ± 6.1	NA	38(74.5%)	0	103	63.7 ± 9.6	55.3 ± 7.8	NA	79 (77.5%)	SA-RFC	Persistent	12 months
Chun et al. (41)	0	101	66.5 ± 9.4	61.5 ± 5.6	21(21)	68(68)	0	101	65.0 ± 9.2	61.5 ± 6.1	12(12)	65(65)	CB-RFC	Paroxysmal or persistent	12 months
Nielsen et al. (44)	1	147	NA	NA	NA	NA	0	141	NA	NA	NA	NA	RFC-MD	Paroxysmal	5 years
Morillo et al. (48)	0	62	54.3 ± 1.3	60.8 ± 7	2 (3.3)	25 (41.0)	0	67	56.3 ± 9.3	61.4 ± 4.8	6 (9.1)	28 (42.4)	RFC-MD	Paroxysmal	24 months
Adiyaman et al. (53)	0	28	55(48–61)	55(50–60)	NA	11 (40.7)	0	24	59(54–66)	55(50-60)	NA	11 (47.8)	SA-RFC	Paroxysmal and Early Persistent	24 months
Castellá et al. (10)	4	63	56.0 ± 7.2	55.5 ± 8.2	NA	NA	1	61	56.1 ± 8.0	57.7 ± 6.8	NA	NA	SA-RFC	Paroxysmal or persistent	7.0 years

In comparisons between RFA and other instrumental strategies, only CBA showed a stable result in random- and fixed-effect models. CBA and RFA did not differ significantly in terms of atrial arrhythmia recurrence risk (OR: 0.76, 95% CI: 0.47–1.22, fixed-effect model; OR: 0.76, 95% CI: 0.11–5.09, random-effects model). Comparisons of SA vs. RFA and AVN + PPM vs. RFA showed unstable results. In SA vs. RFA, SA was associated with a lower risk of atrial arrhythmia (OR: 0.54, 95% CI: 0.30–0.97) in the fixed-effect model, but the difference was not significant using a random-effects model (OR: 0.54, 95% CI: 0.08–3.73). Furthermore, AVN + PPM showed a higher risk of atrial arrhythmia than RFA in the fixed-effect model (OR: 12.81, 95% CI: 1.11–147.52) but no significant difference from the RFA in the random-effects model (OR: 12.81, 95% CI: 0.57–289.38).

Finally, we conducted paired comparisons among CBA, SA, and AVN + PPM. Comparison of SA vs. CBA revealed no difference in atrial arrhythmia recurrence risk (OR: 0.71, 95% CI: 0.34–1.49, fixed-effect model; OR: 0.71, 95% CI: 0.10–5.19, random-effects model). Comparison of AVN + PPM vs. SA showed that AVN + PPM was associated with a greater risk of atrial arrhythmia recurrence (OR: 23.82, 95% CI: 1.97–287.59, fixed-effect model; OR: 23.82, 95% CI: 1.01–559.74, random-effects model); however, comparison of AVN + PPM vs. CBA generated unstable results. In a fixed-effect model, the risk of atrial arrhythmia recurrence in AVN + PPM was higher than that following CBA (OR: 16.89, 95% CI: 1.42–200.41), while using a random-effects model, there was no significant difference (OR: 16.89, 95% CI: 0.73–391.17).

To further validate the effectiveness ranking of the therapies of interest, we conducted replicated calculations of the possibility for each therapy ranking first for the risk of atrial arrhythmia recurrence and constructed SUCRA plots. Figure 2E shows that the area under the SUCRA plot indicated the accumulated possibility of ranking first for recurrence risk; AVN + PPM (AUC = 95.8) showed the highest risk of atrial arrhythmia recurrence, while medication (AUC = 78.2) ranked in second place, and SA (AUC = 5.0) had the lowest risk; RFA (AUC = 47.2) ranked third, and CBA (AUC = 23.8) ranked in fourth place.

Figure 2F presents a funnel plot to illustrate publication bias. Overall publication bias was significant, while for individual comparisons, only CBA vs. RFA and RFA vs. SA showed symmetrical distribution around the funnel plot, indicating low publication bias for those two comparisons.

#### Comparison of All-Cause Re-hospitalization of Patients Undergoing Different Therapies

**FIGURE 3:**
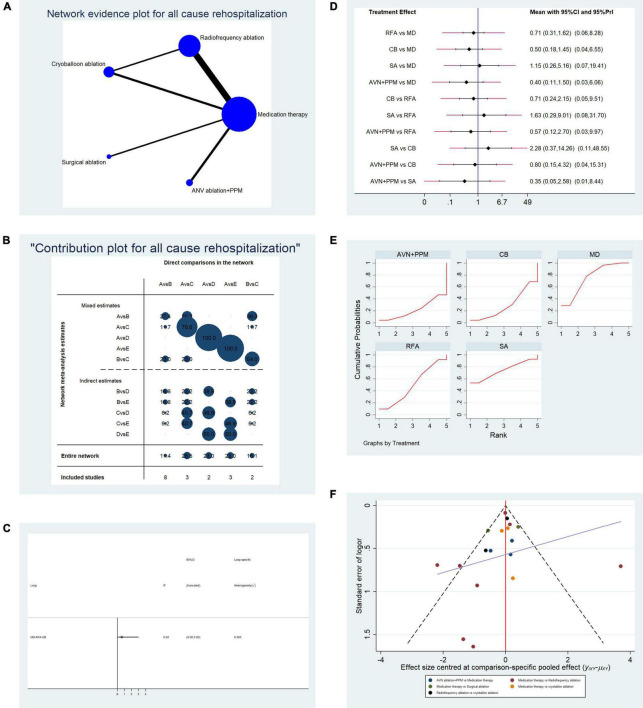
Analysis of risk of all-cause re-hospitalization in patients undergoing five types of treatment for atrial fibrillation. **(A)** Network evidence plot of all-cause re-hospitalization risk. Line thickness represents the number of comparisons between the two arms, and node size represents the sample size of each arm. **(B)** Contribution plot shows the contribution of direct comparisons to the combined comparison of all-cause re-hospitalization risk: A, medication; B, radiofrequency ablation; C, cryoballoon ablation; D, surgical ablation; E, AVN ablation + PPM. **(C)** Ifplot indicates the consistency in each evidence loop for all-cause re-hospitalization risk. **(D)** Pairwise comparison of all-cause re-hospitalization among five therapies for atrial fibrillation. **(E)** SUCRA plot indicates the cumulative possibility of ranking first for the risk of re-hospitalization. **(F)** Funnel plot indicates the risk of publication bias for studies included in the analysis of all-cause re-hospitalization.

Overall, 18 studies reporting all-cause re-hospitalization were included in the pooled analysis. Table 1B shows the basic characteristics of the included studies. The mean age of patients ranged from 53 to 74 years, and most studies had a 12-month follow-up period. Direct comparisons between each treatment by network analysis are shown in Figure 3A. There was only a single concealed evidence loop (MD-RFA-CBA). The contribution of each pairwise comparison to the whole network is illustrated in Figure 3B. Before performing further analyses, we investigated the consistency within the evidence loop. As shown in Figure 3C, the 95% CI of the IF overlapped with the zero-effect line; therefore, we used the structural model to calculate pairwise comparisons. All comparisons, using both random- and fixed-effect models, indicated no significant difference in all-cause re-hospitalization (Figure 3D). Therefore, we next performed replicate calculations to determine the possibility of ranking first for risk of all-cause re-hospitalization. As shown in Figure 3E, drug therapy ranked first for risk of re-hospitalization (SUCRA plot AUC = 75.6), SA ranked second (AUC = 74.1), radiofrequency ablation ranked third (AUC = 49.5), CBA ranked fourth (AUC = 29.1), and AVN + PPM had the lowest risk of all-cause hospitalization (AUC = 21.8). Figure 3F presents a funnel plot to illustrate publication bias. The overall publication bias was significant, and the source may derive from the comparison between MD and RFA; however, the remaining comparisons exhibited almost symmetrical distributions.

**TABLE 1B:** Basic characteristics of studies included in the analysis of the risk of all-cause re-hospitalization.

Author(year)	Arm1			Arm2			Design of study	Type of AF	Follow-up time
	Number of events	Total number	Age	Number of events	Total number	Age			
Blomström-Lundqvist et al. (12)	19	35	65.6 ± 8.8	8	30	69.5 ± 7.9	SA-MD	Permanent	12 months
Helena et al. (13)	35	56	62 ± 7	24	50	59 ± 9	CB-RFC	Paroxysmal or permanent	12 months
Di Biase et al. (14)	67	101	60 ± 11	31	102	62 ± 10	RFC-MD	Permanent	24 months
Buist et al. (15)	58	135	58.2 ± 10.8	33	133	59.7 ± 9.9	CB-RFC	Drug-refractory paroxysmal or early persistent	389 days
Marrouchez et al. (16)	139	175	64	116	164	64	RFC-MD	Paroxysmal or persistent	37.8 months
Forleo et al. (17)	20	35	64.8 ± 6.5	7	35	63.2 ± 8.6	RFC-MD	Paroxysmal or persistent	12 months
Kuck et al. (18)	108	127	67.6 ± 4.6	63	128	67.8 ± 4.8	RFC-MD	Paroxysmal	3 years
Stabile et al. (19)	65	69	62.3 + 10.7	30	68	62.2 ± 9	RFC-MD	Paroxysmal or persistent	12 months
Jaïs et al. (20)	42	55	52.4 ± 11.4	7	53	49.7 ± 10.7	RFC-MD	Paroxysmal	12 months
Kuck et al. (21)	37	84	65 ± 8	13	97	65 ± 8	RFC-MD	Persistent/longstanding persistent	12 months
Haldar et al. (22)	43	60	60.8 ± 10.1	40	54	63.8 ± 8.9	SA-RFC	Long-standing persistent	12 months
Mont et al. (8)	34	48	55 ± 9	39	98	55 ± 9	RFC-MD	Persistent	12-month
Pokushalov et al. (23)	17	32	57 ± 7	6	32	56 ± 7	SA-RFC	PAF/PersAF	12 months
Herrera Siklódy et al. (24)	6	30	56 ± 10	11	30	57 ± 8	CB-RFC	Symptomatic drug-resistant	12 months
Xu et al. (25)	15	35	63.2 ± 9.6	11	39	64.7 ± 9.6	CB-RFC	Non-paroxysmal	12 months
Wang et al. (26)	18	72	51 ± 10	7	66	52 ± 7	SA-RFC	Paroxysmal	12 months
Wilber et al. (27)	57	61	56.1 ± 3.3	54	106	55.5 ± 0.2	RFC-MD	Paroxysmal	9-month
Gu et al. (28)	31	47	55 ± 11	18	48	54 ± 10	SA-RFC	Long-standing persistent	4-year
Andrade et al. (29)	122	149	59.5 ± 10.6	99	154	57.7 ± 12.3	CB-MD	Paroxysmal	12 months
Wazni et al. (30)	60	99	61.6 ± 11.2	34	104	60.4 ± 11.2	CB-MD	Paroxysmal	12 months
Packer et al. (31)	66	71	56 ± 9	82	156	57 ± 9	CB-MD	Symptomatic paroxysmal	12 months
Kuniss et al. (32)	66	104	54.1 ± 13.1	41	94	50.5 ± 13.1	CB-MD	Paroxysmal	12 months
Andrade et al. (7)	53	115	58.6 ± 9.2	111	231	58.9 ± 10.3	CB-RFC	Paroxysmal	12 months
Kuck et al. (33)	87	376	NA	80	374	NA	CB-RFC	Symptomatic paroxysmal	1.5 years
Matta et al. (34)	16	46	59 ± 9	16	46	59 ± 9	CB-RFC	Paroxysmal	12 months
You et al. (35)	14	67	57.7 ± 10.0	22	170	60.2 ± 10.2	CB-RFC	Paroxysmal	12 months
Liu et al. (36)	21	48	55 ± 12	10	50	54 ± 10	RFC-SA	Long-lasting persistent	20 months
Wang et al. (37)	63	70	53.6 ± 10.0	74	140	52.7 ± 9.8	RFC-SA	Persistent	12 months
Osmancik et al. (38)	88	99	71.3 ± 7.9	76	108	69.8 ± 7.9	SA-MD	Paroxysmal, persistent, or long-standing persistent	5 years
DeLurgio et al. (39)	28	51	65.1 ± 6.7	44	102	63.7 ± 9.6	SA-RFC	Persistent and long-standing persistent	12 months
Gunawardene et al. (40)	3	30	57.4 ± 10.5	18	100	62.0 ± 9.5	CB-RFC	Paroxysmal	12 months
Chun et al. (41)	22	100	66.5 ± 9.4	18	100	65.0 ± 9.2	CB-RFC	Paroxysmal and persistent	12 months
Doukas et al. (42)	42	44	67 ± 8	24	45	67.2 ± 9	RFC-MD	Persistent	12 months
Ang et al. (43)	66	67	61 ± 12	66	67	56 ± 10	CB-RFC	Paroxysmal	5 years
Nielsen et al. (44)	87	157	NA	75	149	NA	RFC-MD	Paroxysmal	5 years
Hunter et al. (45)	41	77	61 ± 12	26	78	56 ± 11	CB-RFC	Paroxysmal	12 months
Marshall et al. (46)	2	19	60.3 ± 9.8	12	37	65.2 ± 7.5	ANJPM-MD	Paroxysmal	18 weeks
Wazni et al. (47)	22	35	54 ± 8	4	32	53 ± 8	RFC-MD	Persistent	12 months
Morillo et al. (48)	43	61	54.3 ± 11.3	43	66	56.3 ± 9.3	RFC-MD	Paroxysmal	24 months
Pappone et al. (49)	87	99	57 ± 10	27	99	55 ± 10	RFC-MD	Paroxysmal	4 years
Raatikainen et al. (50)	23	92	56 ± 10	56	110	56 ± 10	RFC-MD	Paroxysmal	24 months
Davtyan et al. (51)	36	44	55.6 ± 12.0	25	45	57.6 ± 8.2	CB-RFC	Paroxysmal	12 months
Poole et al. (52)	317	629	68	444	611	68	RFC-MD	Paroxysmal, persistent, or long-standing persistent	5 years
Adiyaman et al. (53)	12	27	59 (54–66)	15	23	55 (48–61)	SA-RFC	Paroxysmal or early persistent	24 months
Gillinov et al. (54)	72	102	69.4 ± 10.0	39	106	69.7 ± 10.4	SA-MD	Persistent or long-standing persistent	12 months
Alhede et al. (55)	85	132	54 ± 10	68	128	56 ± 9	RFC-MD	Paroxysmal	24 moths
Castellá et al. (10)	55	63	56.0 ± 7.2	34	61	56.1 ± 8.0	SA-RFC	Paroxysmal AND persistent	7 years
Gallagher et al. (56)	38	50	NA	33	49	NA	CB-RFC	Long-standing persistent	12 months

### Evaluation of Safety Endpoints

#### Evaluation of the Risk of Ischemic Stroke Among Patients Undergoing the Five Therapies

**FIGURE 4:**
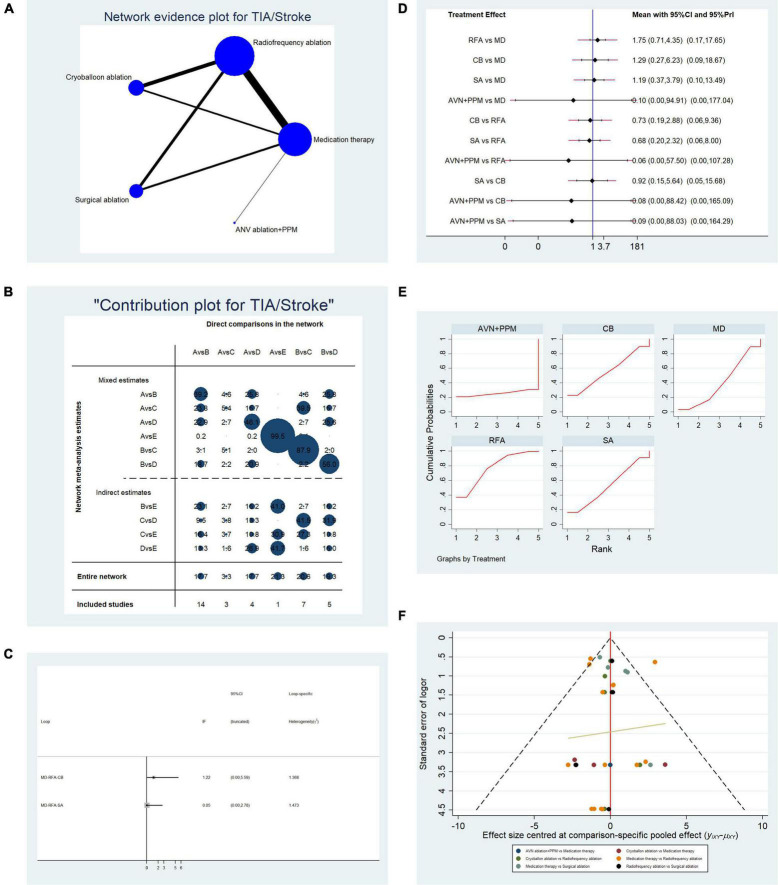
Analysis of risk of stroke/transient ischemic attack (TIA) in patients undergoing five types of treatment for atrial fibrillation. **(A)** Network evidence plot for the investigation of stroke/TIA risk. Line thickness represents the number of comparisons between the two arms, and node size represents the sample size of each arm. **(B)** Contribution plot showing the contribution of direct comparisons to the combined comparison of stroke/TIA risk: A, medication; B, radiofrequency ablation; C, cryoballoon ablation; D, surgical ablation; E, AVN ablation + PPM. **(C)** Ifplot indicates the consistency in each evidence loop for stroke/TIA risk. **(D)** Pairwise comparison of stroke/TIA risk among five therapies for atrial fibrillation. **(E)** SUCRA plot indicates the cumulative possibility of ranking first for stroke/TIA risk. **(F)** Funnel plot indicates the risk of publication bias of studies included in the analysis of stroke/TIA risk.

Overall, 34 studies that reported the relevant endpoint (stroke/TIA) were included in the pooled analysis. Table 2A presents the basic characteristics of the included studies. We included left atrium size in this study since a recent study demonstrated that left atrium size is associated with the risk of ischemic stroke (70). In most studies, there was no difference in left atrium size between the two arms. A network plot representing the number of direct comparisons was constructed (Figure 4A), and the proportion contribution of each direct comparison to the mixed comparison was determined (Figure 4B). Figure 4C presents the results of an evidence consistency test. In the evidence loops, MD-RFA-CBA and MD-RFA-SA, the 95% CI of the IF overlapped with the zero-effect line. Thus, the NMA consistency was acceptable. Subsequent pairwise analysis revealed no difference in the occurrence of ischemic cerebral vascular event (CVE) (Figure 4D). Thus, we adopted a frequentist-based method by replicating the calculation of the accumulated possibility of ranking first in terms of CVE risk. SUCRA plots (Figure 4E) showed that RFA therapy was associated with the highest risk of developing CVE (AUC = 76.7), CBA ranked second (AUC = 55.7), SA ranked third (AUC = 52.3), medication ranked fourth (AUC = 39.8), and AVN + PPM therapy ranked lowest (AUC = 25.5). Figure 4F illustrates the risk of publication bias.

**TABLE 2A:** Basic characteristics of studies included in the analysis of the risk of stroke/TIA.

Author(year)	Arm1			Arm2			Design	Type of AF	Follow-up time
	Number of events	Total number	Age	Number of events	Total number	Age			
Brignole et al. (11)	13	52	72 ± 9	5	50	71 ± 12	ANJPMA-MD	Permanent	16 months
Brignole et al. (57)	13	34	72 ± 9	9	32	72 ± 9	ANJPMA-MD	Persistent	12 month
Brignole et al. (58)	28	70	74 ± 9	11	63	72 ± 11	ANJPMA-MD	Permanent	29 months
Marrouche et al. (16)	122	184	64	114	179	64	RFC-MD	Paroxysmal or persistent	37.8 months
Forleo et al. (17)	12	35	64.8 ± 6.5	3	35	63.2 ± 8.6	RFC-MD	Paroxysmal or persistent	12 months
Mont et al. (8)	3	48	55 ± 9	2	98	55 ± 9	RFC-MD	Persistent	12-month
Xu et al. (25)	15	35	63.2 ± 9.6	8	39	64.7 ± 9.6	CB-RFC	Non-paroxysmal	12-month
Andrade et al. (29)	43	149	59.5 ± 10.6	33	154	57.7 ± 12.3	CB-MD	Paroxysmal	12 months
Wazni et al. (30)	43	99	61.6 ± 11.2	31	104	60.4 ± 11.2	CB-MD	Paroxysmal	12 months
Packer et al. (31)	5	163	56 ± 9	2	82	57 ± 9	CB-MD	Paroxysmal AF	12 months
Kuck et al. (59)	156	376	NA	122	374	NA	CB-RFC	Paroxysmal	1000 days
Poole et al. (52)	637	1096	68 (64-73)	573	1108	68 (64-73)	RFC-MD	Paroxysmal or persistent	60 months
Osmancik et al. (38)	38	99	71.3 ± 7.9	33	108	69.8 ± 7.9	SA-MD	Paroxysmal, persistent, or long-standing persistent	60 months
Noseworthy et al. (60)	4	21824	73.4 ± 10.2	4	704	65.9 ± 10.2	RFC-MD	NA	1.8 years
Cosedis Nielsen et al. (61)	2	149	54 ± 10	0	147	56 ± 9	RFC-MD	Paroxysmal	24 months
Wazni et al. (47)	19	35	54 ± 8	3	32	53 ± 8	RFC-MD	Symptomatic	12 months
Raatikainen et al. (50)	1	93	56 ± 10	0	111	56 ± 10	RFC-MD	Paroxysmal	24 months
Gillinov et al. (54)	54	127	69.4 ± 10.0	77	133	69.7 ± 10.4	SA-MD	Persistent or long-standing persistent	12 months

#### Evaluation of All-Cause Mortality Among Patients Undergoing the Five Therapies

**FIGURE 5:**
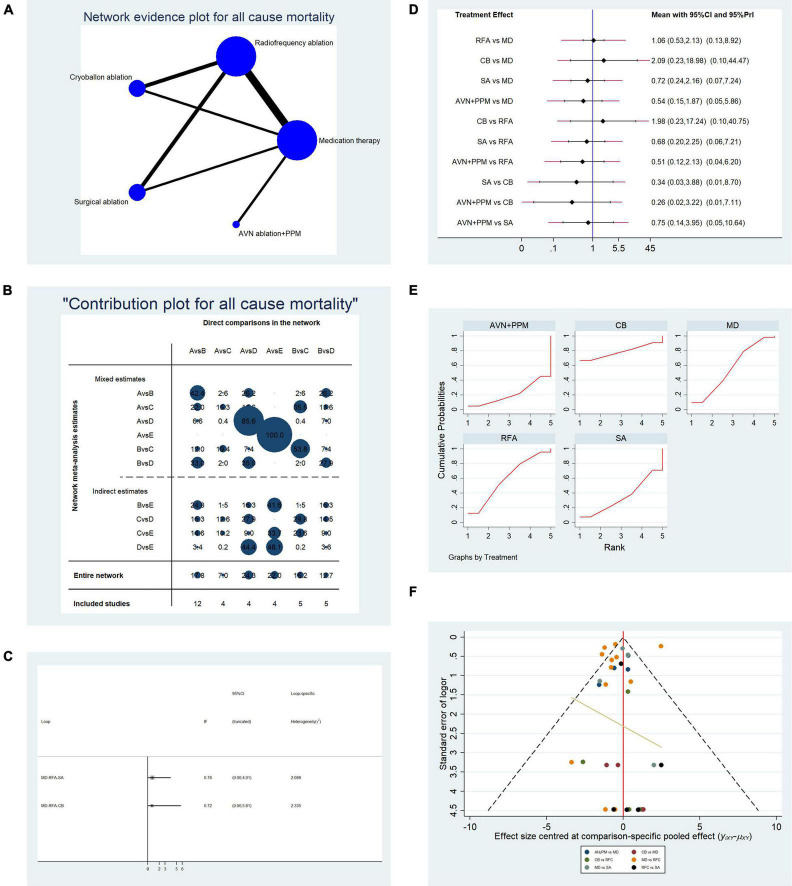
Analysis of risk of all-cause mortality in patients undergoing five types of treatment for atrial fibrillation. **(A)** Network evidence plot for all-cause mortality risk. Line thickness represents the number of comparisons between the two arms, and node size represents the sample size of each arm. **(B)** Contribution plot shows the contribution of direct comparisons to the combined comparison for risk of all-cause mortality: A, medication; B, radiofrequency ablation; C, cryoballoon ablation; D, surgical ablation; E, AVN ablation + PPM. **(C)** Ifplot indicates the consistency in each evidence loop for investigation of all-cause mortality. **(D)** Pairwise comparison of all-cause mortality among five therapies for the treatment of atrial fibrillation. **(E)** SUCRA plot indicates the cumulative possibility of ranking first for the risk of all-cause mortality. **(F)** Funnel plot indicates the risk of publication bias for studies included in the analysis of all-cause mortality.

Overall, data from 34 studies that reported all-cause mortality were pooled. Table 2B presents the basic characteristics of the included studies. Since mortality is associated with multiple factors, we also included duration of AF, left ventricular ejection fraction, and New York Heart Association classification (Table 2B). As shown in Table 2B, no significant difference in confounding factors was detected between the two arms. Figure 5A presents a network plot illustrating the number of direct comparisons, and the proportion contribution of each direct comparison to the mixed comparison is shown in Figure 5B. The results of consistency testing are shown in Figure 5C and revealed that the evidence loops MD-RFA-CBA and MD-RFA-SA had IF 95% CI values that overlapped with the zero-effect line. Thus, the consistency of the NMA was acceptable. Subsequent pairwise analysis revealed no differences in the occurrence of all-cause mortality. As shown in Figure 5D, no significant differences in all-cause mortality were detected using either random- or fixed-effect models. Thus, we adopted a frequentist-based model, by replicating calculations of the accumulated possibility of ranking first for all-cause mortality. As shown in the SUCRA plots in Figure 5E, CBA ablation ranked first for all-cause mortality (AUC = 78.0), RFA ranked second (AUC = 59.9), medication ranked third (AUC = 56.6), SA ranked third (AUC = 34.9), and AVN + PPM ranked lowest (AUC = 21.0). A funnel plot illustrating publication bias is shown in Figure 5F.

**TABLE 2B:** Basic characteristics of studies included in the analysis of the risk of all-cause mortality.

Author(year)	Arm1				Arm2				Design	Type of AF	Follow-up time
	Number of events	Total number	Age	LA size (mm)	Number of events	Total number	Age	LA size (mm)			
Schmidt et al. (62)	8	33	63 ± 10	41 ± 6	6	33	66 ± 10	40 ± 5	CB-RFC	Drug-refractory paroxysmal	24–48 h
Blomström-Lundqvist et al. (12)	2	35	65.6 ± 8.8	58 ± 7	4	34	69.5 ± 7.9	61 ± 11	SA-MD	Permanent	12 months
David (63)	1	70	68.3 ± 10	NA	1	69	61.9 ± 9.08	NA	CB-RFC	Persistent or paroxysmal	12 months
Buist et al. (15)	1	136	58.2 ± 10.8	NA	0	133	59.7 ± 9.9	NA	CB-RFC	Persistent or paroxysmal	389 days
Brignole et al. (57)	1	34	72 ± 9	49 ± 6	0	32	72 ± 9	52 ± 10	ANJPM-MD	Persistent	12 months
Marrouche et al. (16)	11	184	64	49.5	5	179	64	48	RFC-MD	Paroxysmal or persistent	37.8 months
Forleo et al. (17)	0	35	64.8 ± 6.5	45.2 ± 5.2	0	35	63.2 ± 8.6	44.3 ± 5.6	RFC-MD	Paroxysmal or persistent	12 months
Kuck et al. (18)	1	108	67.6 ± 4.6	43.4 ± 5.6	0	102	67.8 ± 4.8	42.1 ± 6.1	RFC-MD	Paroxysmal	3 years
Stabile et al. (19)	1	69	62.3 ± 10.7	45.4 ± 5.5	1	68	62.2 ± 9	46 ± 5	RFC-MD	Paroxysmal or persistent	12 months
Haldar et al. (60)	1	60	60.8 ± 10.1	44.6 ± 6	1	55	63.8 ± 8.9	44.7 ± 5.8	SA-RFC	Long-standing persistent	12 months
Mont et al. (8)	0	48	55 ± 9	42.7 ± 5.1	0	98	55 ± 9	41.3 ± 4.6	RFC-MD	Persistent	12 months
Pokushalov et al. (23)	1	32	57 ± 7	45 ± 7	0	32	56 ± 7	46 ± 5	SA-RFC	Paroxysmal and persistent	12 months
Wilber et al. (27)	0	61	56.1 ± 3.2	40.5 ± 1.5	0	106	55.5 ± 1.8	40 ± 1.1	RFC-MD	Persistent	12 months
Gu et al. (28)	0	47	55 ± 11	60.4 ± 10.0	1	48	54 ± 10	61.7 ± 9.6	SA-MD	Long-standing persistent	4 years
Andrade et al., (29)	1	149	59.5 ± 10.6	38.1 ± 6.5	0	154	57.7 ± 12.3	39.5 ± 5.0	CB-MD	Paroxysmal	12 months
Wazni et al. (30)	0	99	61.6 ± 11.2	38.2 ± 5.4	1	104	61.6 ± 11.2	38.7 ± 5.7	RFC-MD	Paroxysmal	12 months
Packer et al. (31)	0	82	56 ± 9	41 ± 6	7	163	57 ± 9	40 ± 5	CB-MD	Paroxysmal	12 months
Kuniss et al. (32)	0	111	54.1 ± 13.4	47.7 ± 6.3	1	107	50.5 ± 13.1	46.8 ± 8.2	CB-MD	Paroxysmal	12 months
Kuck et al. (33)	2	376	60.1 ± 9.2	40.6 ± 5.8	2	374	59.9 ± 9.8	40.8 ± 6.5	CB-RFC	Paroxysmal	1.5 years
Luik et al. (64)	0	159	60 (54, 67)	NA	0	156	61 (54, 66)	NA	CB-RFC	Paroxysmal	12 months
Packer et al. (65)	7	1096	67 (62, 72)	NA	3	1108	68(62, 72)	NA	RFC-MD	Persistent, paroxysmal and Long-standing persistent	48.5 months
Liu et al. (36)	1	49	55 ± 12	60.4 ± 10.7	0	50	54 ± 10	61.3 ± 9.8	SA-RFC	Long-lasting persistent	20 months
Osmancik et al. (38)	14	99	71.3 ± 7.9	47.0 ± 6.1	6	108	69.8 ± 7.9	48.6 ± 7.3	SA-MD	Paroxysmal, persistent, or long-standing	5 years
Noseworthy et al. (60)	5	21824	73.4 ± 10.2	NA	5	704	65.9 ± 10.2	NA	RFC-MD	Long-standing persistent	1.8 years
Chun et al. (41)	1	100	66.5 ± 9.4	39.8 ± 5.2	0	100	65.0 ± 9.2	39.1 ± 5.3	CB-RFC	Paroxysmal and persistent	12 months
Doukas et al. (42)	1	48	67 ± 8	60 ± 11	2	49	67.2 ± 9	58 ± 7	RFC-MD	Persistent	12 months
Cosedis Nielsen et al. (61)	1	148	54 ± 10	40 ± 5	2	146	56 ± 9	40 ± 6	RFC-MD	Paroxysmal	24 months
Morillo et al. (48)	0	61	54.3 ± 11.7	43 ± 5	0	66	56.3 ± 9.3	40 ± 5	RFC-MD	Paroxysmal	24 months
Pappone et al. (49)	0	22	57 ± 10	NA	1	186	55 ± 10	NA	RFC-MD	Paroxysmal	4 years
Raatikainen et al. (50)	0	92	56 ± 10	40 ± 5	2	110	56 ± 10	40 ± 5	RFC-MD	Paroxysmal	24 months
Sørensen et al. (66)	1	51	62 (55–69)	40.4 ± 5.3	0	50	60(55-65)	40.2 ± 5.2	CB-RFC	Paroxysmal	6 months
Adiyaman et al. (53)	0	23	59 (54–66)	40 (38–44)	0	27	55(48-61)	39(37–42)	SA-RFC	Paroxysmal and Early Persistent	2 years
Gillinov et al. (54)	2	127	69.4 ± 10.0	NA	4	133	69.7 ± 10.4	NA	SA-MD	Persistent or long-standing persistent	12 months
Castellá et al. (10)	6	63	56.0 ± 7.2	43.2 ± 4.8	6	61	56.1 ± 8.0	42.5 ± 6.5	SA-RFC	Paroxysmal or persistent	7 years

#### Evaluation of Cardiovascular Mortality Among Patients Receiving Five Therapies

**FIGURE 6:**
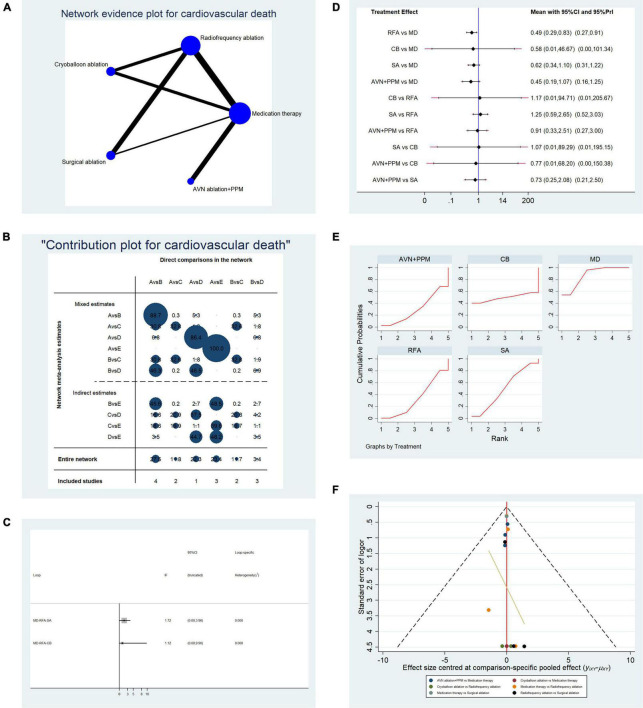
Analysis of risk of cardiovascular death in patients undergoing five types of treatment for atrial fibrillation. **(A)** Network evidence plots for investigation of cardiovascular death. Line thickness represents the number of comparisons between the two arms, and node size represents the sample size of each arm. **(B)** Contribution plot shows the contribution of direct comparisons to combined comparison in the investigation of cardiovascular death: A, medication; B, radiofrequency ablation; C, cryoballoon ablation; D, surgical ablation; E, AVN ablation + PPM. **(C)** Ifplot indicates the consistency in each evidence loop for cardiovascular mortality. **(D)** Pairwise comparison of cardiovascular mortality among five therapies for atrial fibrillation. **(E)** SUCRA plot indicates the cumulative possibility of ranking first for the risk of cardiovascular mortality. **(F)** Funnel plot indicates the risk of publication bias for studies included in the analysis of cardiovascular mortality.

Overall, data from 15 studies reporting cardiovascular mortality were pooled. Table 2C presents the basic characteristics of included studies. A total of 2,861 patients were included in the pooled analysis, with cardiovascular mortality occurring in 161 patients. Figure 6A presents a network plot representing the number of direct comparisons, and Figure 6B presents the proportion contributions of each direct comparison to the overall contribution. Figure 6C presents evidence consistency test results and revealed that, for the evidence loops, MD-RFA-CBA and MD-RFA-SA, the IF 95% CI values overlapped with the zero-effect line; thus, the consistency of the NMA was acceptable. Subsequent pairwise analysis revealed that RFA had a significantly lower risk of cardiovascular mortality than medication therapy (OR: 0.49, 95% CI: 0.29–0.83, fixed-effect model; OR: 0.49, 95% CI: 0.27–0.9, random-effects model); however, no other comparisons showed significant differences (Figure 6D). Therefore, we calculated the possibility of each therapy ranking first for risk of cardiovascular death. Analysis of the SUCRA plots (Figure 6E) indicated that medication has the highest risk of cardiovascular death (AUC = 88.1), with SA second (AUC = 50.6), CBA third (AUC = 48.3), RFA fourth (AUC = 33.2), and AVN + PPM ranked lowest (AUC = 29.8). Figure 6F presents the publication bias analysis. As shown in the funnel plot, the distribution of comparisons was almost symmetrical; thus, the publication bias was low.

**TABLE 2C:** Basic characteristics of studies included in the analysis of the risk of cardiovascular mortality.

Author(year)	Arm1						Arm2						Design	Type of AF	Follow up time
	Number of events	Total number	age	LVEF%	NYHA ≥ III Number (percent)	AF Duration	Number of events	Total number	Age	LVEF%	NYHA ≥ III Number (percent)	AF Duration			
Brignole et al. (11)	6	52	72 ± 9	40 ± 12	34 (65)	18 (8–43)	2	50	71 ± 12	41 ± 12	32 (64)	13 (8–36)	ANJPM-MD	Permanent	16 months
Blomström-Lundqvist et al. (12)	0	35	65.6 ± 8.8	57 ± 12	24 (68.6)	69 ± 74	1	34	69.5 ± 7.9	53.6 ± 9.1	20(66.7)	49 ± 57	SA-MD	Permanent	12 months
Di Biase et al. (14)	18	100	60 ± 11	30 ± 8	NA	8.4 ± 4.1	8	100	62 ± 10	29 ± 5	NA	8.6 ± 3.2	RFC-MD	Permanent	24 months
Buist e al. (15)	0	136	58.2 ± 10.8	NA	NA	4.8 ± 5.6 YEARS	0	133	59.7 ± 9.9	NA	NA	5.1 ± 6.1	CB-RFC	Persistent or paroxysmal	389 days
Brignole et al. (57)	4	34	72 ± 9	NA	NA	4.1 ± 5	3	32	72 ± 9	NA	NA	5.7 ± 6.9	ANJPM-MD	Persistent	12 month
Brignole et al. (58)	20	70	74 ± 9	NA	49 (70)	18(8–38)	7	63	72 ± 11	NA	42 (67)	19 (8–48)	ANJPM-MD	Permanent	2 years
Marrouche et al. (16)	46	184	64	31.5	51(28)	NA	24	179	64	32.5	53(31)	NA	RFC-MD	Paroxysmal or persistent	37.8 months
Stabile et al. (19)	2	69	62.3 ± 10.7	57.9 ± 5.8	NA	85.2 ± 70.8	1	68	62.2 ± 9	59.1 ± 6.7	NA	61.2 ± 46.8	RFC-MD	Paroxysmal or persistent	12 months
Jaïs et al. (20)	2	59	52.4 ± 11.4	65.6 ± 7.2	NA	NA	0	53	49.7 ± 10.7	63.1 ± 11.0	NA	NA	RFC-MD	Paroxysmal	12 months
Kuck et al. (21)	8	100	65 ± 8	24.8 ± 8.8	45 (62)	NA	8	98	65 ± 8	27.8 ± 9.5	40 (59)	NA	RFC-MD	Persistent	12 months
Haldar (22)	0	60	60.8 ± 10.1	55.2 ± 8.9	NA	19.5(15–29.2)	1	55	63.8 ± 8.9	58.8 ± 8.7	NA	25(19–35.5)	SA-RFC	Long-standing persistent	12 months
Mont et al. (8)	0	48	55 ± 9	60.8 ± 9.7	0	NA	0	98	55 ± 9	61.1 ± 8.8	3 (3.1)	NA	RFC-MD	Persistent	12 months
Andrade et al. (29)	0	149	59.5 ± 10.6	59.8 ± 7.6	NA	1 (0–4)	0	154	57.7 ± 12.3	59.6 ± 7.0	NA	1 (0–3)	CB-MD	Paroxysmal	12 months
Packer et al. (31)	0	82	56 ± 9	61 ± 6	0	NA	1	163	57 ± 9	60 ± 6	0	NA	CB-MD	Paroxysmal	12 months
Kuniss et al. (32)	0	111	54.1 ± 13.4	63.7 (5.4)	NA	8.4 ± 18	0	107	50.5 (13.1)	62.8 (5.4)	NA	9.6 ± 25.2	CB-MD	Paroxysmal	12 months
Andrade et al. (7)	0	115	58.6 ± 9.2	59.1 ± 6.6	NA	4(2.0-10.0)	0	231	58.9 ± 10.3	59.3 ± 6.8	NA	4.0(2.0-15.0)	CB-RFC	Paroxysmal	12 months
Kuck et al. (33)	0	376	60.1 ± 9.2	NA	1	4.7 ± 5.3	2	374	59.9 ± 9.8	NA	0	4.6 ± 5.1 YEARS	CB-RFC	Paroxysmal	1.5 years
Luik et al. (64)	1	159	60(54-67)	NA	NA	NA	1	150	61 (54, 66)	NA	NA	NA	CB-RFC	Paroxysmal	12 months
Packer et al. (65)	67	1092	67(62-72)	NA	400 (36.7)	NA	58	1006	68 (62-72)	NA	376 (34.3)	NA	RFC-MD	Paroxysmal or persistent	48.5 months
Abreu Filho et al. (67)	4	28	50.7 ± 9.7	62.8 ± 9.2	28	43.8 ± 8.5	1	42	55.4 ± 12.8	66. ± 10.5	42	66.1 ± 57.4	SA-MD	Permanent	12 months
Wang et al. (37)	0	70	53.6 ± 10.0	61.2 ± 6.4	28(40)	33.7 ± 20.9	1	140	52.7 ± 9.8	61.3 ± 6.7	59(42)	32.9 ± 3.9	SA-MD	Chronic	12 months
Osmancik et al. (38)	39	99	71.3 ± 7.9	49.6 ± 12.5	NA	39.5 ± 50.9	32	108	69.8 ± 7.9	52.2 ± 11.2	NA	39.2 ± 53.6	SA-MD	Paroxysmal, persistent, or long-standing persistent	5 years
Noseworthy et al. (60)	53	282366	73.4 ± 10.2	NA	NA	NA	26	7465	65.9 ± 10.2	NA	NA	NA	RFC-MD	NA	1.8 years
DeLurgio et al. (39)	0	51	65.1 ± 6.7	55.7 ± 6.1	NA	4.5 ± 4.7	0	102	63.7 ± 9.6	55.3 ± 7.8	NA	4.4 ± 4.8	SA-RFC	Persistent	12 months
Chun et al. (41)	0	100	66.5 ± 9.4	61.5 ± 5.6	NA	NA	0	100	65.0 ± 9.2	61.5 ± 6.1	NA	NA	CB-RFC	Paroxysmal or persistent	12 months
Doukas et al. (42)	4	48	67 ± 8	NA	NA	46.7 ± 64.3	3	49	67.2 ± 9	NA	NA	57 ± 55.1	RFC-MD	Persistent	12 months
Nielsen et al. (44)	7	146	NA	NA	NA	NA	5	140	NA	NA	NA	NA	RFC-MD	Paroxysmal	5 years
Morillo et al. (48)	0	61	54.3 ± 11.7	60.8 ± 7.0	NA	NA	0	66	56.3 ± 9.3	61.4 ± 4.8	NA	NA	RFC-MD	Paroxysmal	24 months
Raatikainen et al. (50)	1	92	56 ± 10	64 ± 7	NA	NA	3	110	56 ± 10	63 ± 10	NA	NA	RFC-MD	Paroxysmal	24 months
Adiyaman et al. (53)	0	27	55(48–61)	55(50–60)	NA	3.9(1.5–8.0)	0	23	59(54–66)	55(50–60)	NA	3.6(1.6–8.7)	SA-RFC	Paroxysmal and Early Persistent	24 months
Sugihara et al. (68)	0	49	NA	NA	NA	NA	0	20	NA	NA	NA	NA	SA-RFC	Paroxysmal	12 months
Gillinov et al. (54)	11	127	69.4 ± 10.0	56.5 ± 7.7	62(49.2)	NA	9	113	69.7 ± 10.4	55.1 ± 7.6	56 (42.1)	NA	SA-MD	Persistent or long-standing persistent	12 months
Weerasooriya et al. (69)	1	50	67.9 ± 9	57 ± 14	NA	NA	2	49	68 ± 8.5	55 ± 16	NA	NA	ANJPM-MD	Permanent	12 months
Castellá et al. (10)	5	63	56.0 ± 7.2	55.5 ± 8.2	NA	NA	4	61	56.1 ± 8.0	57.7 ± 6.8	NA	NA	SA-RFC	Paroxysmal or persistent	7.0 years

## Discussion

Analysis of AF recurrence rate only showed a significant and robust difference between AVN + PPM and SA (OR: 23.82, 95% CI: 1.97–287.95, fixed-effect model; OR: 23.82, 95% CI: 1.01–559.74, random-effects model); however, comparisons of other instrumental therapies generated unstable results, with the risk estimates for RFA, CBA, and SA lower than that for traditional drug therapy in fixed-effect models, but not significant using random-effects models. By contrast, in comparisons between instrumental therapies, CBA showed no significant difference to RFA or SA in terms of atrial arrhythmia recurrence, with robust results generated using both the random- and fixed-effect models. Although SA and AVN + PPM differed from RFA, the results were unstable using a random-effects model. To further rank the therapies, we generated SUCRA plots and found that AVN + PPM ranked first in terms of arrhythmia recurrence, while SA had the best efficacy in reducing the risk of atrial arrhythmia. Although AVN + PPM is a good method for rhythm control in patients with AF, this type of therapy is limited to non-responders to pharmaceutical rhythm control. Furthermore, most patients need PPM implantation after AVN ablation and continuous pacing is needed; thus, continuous monitoring and programming of the pacemaker are required, increasing the economic burden of healthcare.

In an analysis of all-cause re-hospitalization, pairwise comparisons among the five therapies revealed no significant difference, with stable results generated using both random- and fixed-effect models. Therefore, we calculated the likelihood of each therapy ranking first in risk for all-cause re-hospitalization. Medication was associated with the highest risk of re-hospitalization, SA ranked second, and AVN + PPM ranked lowest, with CBA and RFA having intermediate ranks. Pairwise comparisons of the risk of ischemic stroke generated similar results. AVN + PPM also ranked lowest for risk of ischemic stroke according to frequentist analysis, while RFA ranked the highest risk. CBA and SA showed similar risks, and medication appeared to be associated with lower risk.

To evaluate the safety of the five therapies, we selected ischemic cerebral vascular events, including stroke and TIA, as one safety event endpoint. AF increases the risk of ischemic stroke by disturbing hemodynamic stasis in the left atrium (71). Thus, resuming a normal atrial electrical activity rhythm is crucial to the prevention of ischemic stroke, and ischemic stroke onset is an appropriate endpoint for the evaluation of AF therapy safety. High mortality rate is another characteristic of AF; a 2016 study with 134,046 patient-years of follow-up revealed that all-cause mortality was 9% in patients diagnosed with AF (72). Moreover, the Framingham Heart Study reported that the risk of death increases by 1.5-fold and 1.9-fold in men and women diagnosed with AF, respectively (73). Thus, all-cause mortality is an appropriate safety endpoint for therapy evaluation. Furthermore, there is substantial evidence that AF is an independent risk factor for sudden cardiac death (74).

Pairwise comparisons showed no significant difference in all-cause mortality between the five therapies; however, frequentist analysis indicated that CBA led to the highest all-cause mortality, while AVN + PPM ranked the lowest for risk of re-hospitalization. Regarding cardiovascular mortality, RFA clearly led to a lower risk of cardiovascular mortality in pairwise comparisons using both random- (OR: 0.49, 95% CI: 0.27–0.9) and fixed-effect (OR: 0.49, 95% CI: 0.29–0.83) models. No significant differences were detected using either random- or fixed-effect models for any other comparisons. Subsequent frequentist analysis revealed that AVN + PPM ranked lowest for risk of cardiovascular mortality, while medication ranked highest.

The main goal of this study was to rank the five therapies for the endpoints of interest. In particular, our analysis indicated that AVN + PPM had advantages in reducing the risk for most endpoint events; however, the recurrence rate of atrial arrhythmia was highest in patients undergoing AVN + PPM among the therapies. There are several potential explanations for this result. First, the benefits of AVN + PPM may derive from the improved ejection fraction achieved by this approach, which is higher than that for AVN ablation + CRT (11). Furthermore, a similar previous study showed that the adoption of ablation combined with pacemaker implantation can reduce all-cause mortality relative to PPM alone (75). Regarding the high atrial arrhythmia recurrence rate, we speculate that ablation of the AVN node stops the dysregulated atrial rhythm from passing to the ventricle, while the mechanism underlying the atrial arrhythmia is not addressed and thus remains. Implantation of a pacemaker may provide a bridge between the atrium and ventricle, facilitating the transduction of atrial rhythm to the ventricle.

**FIGURE 7:**
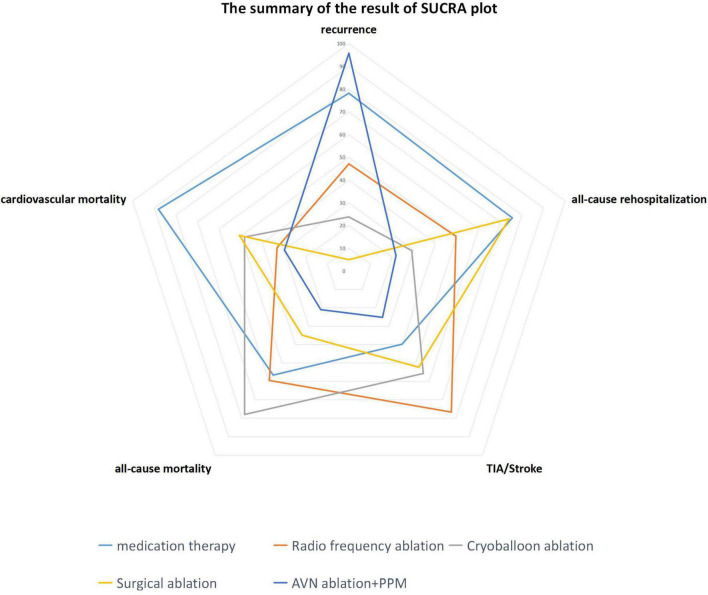
Radar plot indicating the overall risk of efficacy and safety endpoints based on the area under the curve of SUCRA plots.

As shown in the radar plot in Figure 7, medication had the greatest risk of safety and efficacy events, consistent with most previous studies. Thus, this study confirms the advantages of instrumental therapy over traditional therapy for AF; however, our study is the first to also compare efficacy and safety risk factors among four other instrumental therapies. As a more invasive therapy, SA showed the best performance among the five therapies. Furthermore, relative to RFA and CBA, there was a lower risk of safety and efficacy events in patients undergoing SA, illustrated by a smaller area in the radar plot (Figure 7). One possible explanation for this result is that SA is a technique operated on the epicardium, and improved penetration of the epicardium by bipolar RFA leads to persistent conduction block (23). In addition, SA is always accompanied by left appendage resection, which may also contribute to freedom from AF recurrence (76). RFA has become a primary approach for control of heart rhythm and is widely adopted; however, in our analysis, patients who received RFA showed the highest risk of ischemic stroke among the therapies. This result is contrary to the findings of a previous meta-analysis, which only compared RFA and medication therapy. One possible reason for this discrepancy is that the previous meta-analysis only involved one RCT and investigated only one comparison (77), while in our study we used NMA to conduct more comparisons simultaneously. Thus, our analysis included more co-variants that could affect the final result and may represent a better approximation of the real-world situation. In pairwise comparisons, our results indicated that medication and RFA did not differ significantly in terms of stroke risk, as indicated in the CABANA trial (78). As an improvement of traditional RFA therapy, CBA is expected to increase the efficiency of pulmonary vein isolation, as it only requires a single procedure (79). Recent studies show that, with regard to safety and efficacy, CBA is not inferior to RFA (7). Here, our study demonstrated that the overall risk of occurrence of efficacy and safety endpoints is slightly lower following CBA than RFA, as shown in the radar plot. Nevertheless, all-cause mortality was highest in patients undergoing CBA among the five therapies, possibly because of the potential complications of CBA, such as phrenic nerve injury (80) and pulmonary vein complications (81).

The strengths of this study include the comprehensive, and systematic literature search was conducted. Furthermore, to better evaluate treatment safety and efficacy, we adopted multiple endpoints, including atrial arrhythmia recurrence, all-cause re-hospitalization, ischemic stroke, all-cause mortality, and cardiovascular mortality, which likely diminished publication bias. Moreover, we conducted NMA to make comparisons that have not previously been analyzed. Furthermore, we identified confounding factors that may have affected the final results; however, these confounding factors did not differ significantly in most of the studies. This study also had some limitations. Various types of AF were adopted in the inclusion criteria, which are a major source of heterogeneity; however, this type of design, involving heterogeneous types of AF, may better imitate real-world conditions and increase the likelihood that our conclusions are valid in various situations. As for the pooling method, most of the pairwise comparisons conducted generated non-significant results; thus, most of our conclusions are derived from SUCRA plots. SUCRA plots indicate the percentage effectiveness of each treatment, accounting for all possible rankings and uncertainties in treatment effects; however, we replicated the calculations 10,000 times, as random effects can be eliminated with sufficient rounds of replication. Furthermore, particularly in the evaluation of atrial arrhythmia recurrence, we observed that the results of some comparisons were unstable under fixed- and random-effects models. A potential explanation for this could be that, within the evidence loop, the contribution of direct comparisons to the combined comparison was too small. For example, the differences between instrumental therapies (RFA, CBA, and SA) and traditional medication were statistically significant in the fixed-effect model, while the findings lacked significance when analyzed using a random-effects model. In network contribution plots, the contributions of direct comparisons to combined comparison were 19, 29.8, and 63.4%, respectively. Although the contribution of direct comparison was high in SA vs. MD, publication bias remained, leading to the heterogeneity of the involved studies. Also, the width of calculated 95% CI values was relatively large, due to the presence of zero events. Nevertheless, the frequentist analysis helped to overcome the shortcomings of pairwise comparisons. Regarding the analysis of publication bias of individual studies, evaluation of the source of bias (Supplementary Figures 1, 2) revealed that most bias derived from the blinding method, and this type of bias is inevitable, as the inherent characteristics of studies investigating this topic make it particularly challenging to blind patients to therapy type. Furthermore, confounding factors such as age, anti-coagulation drugs, and CHADSVAS score were not discussed, since it is challenging to integrate such factors into the comparison network; hence, subgroup analyses are not generally conducted in NMA. This may be because subgroup analysis would destroy the original evidence loop, meaning that arms present in the original evidence loop would be eliminated from the subgroup analysis and the final results could be affected by both confounding factors and the novel evidence loop. Thus, the effect of confounding factors on the final results was high. Furthermore, the age of patients involved in studies reported in the literature may be a source of selection bias, since older patients are reluctant to choose interventional therapy; however, the RCT studies included had balanced baseline age in study arms.

## Conclusion

In this NMA, we first compared efficacy and safety endpoints among five therapies: medication, RFA, CBA, SA, and AVN + PPM. In pairwise comparisons, AVN + PPM clearly showed a higher risk of atrial arrhythmia recurrence, while RFA showed a significantly lower risk of cardiovascular mortality than medication. The results of frequentist analysis using SUCRA plots indicated that AVN + PPM performs best, in terms of the risk of efficacy and safety endpoints, while medication had the worst performance in reducing these endpoints. Of the remaining three therapies, SA performed best in reducing recurrence, while RFA and CBA had almost the same overall risk of efficacy and safety endpoints; however, SA still has advantages over these two strategies in reducing the overall risk of such events.

## Data Availability Statement

The original contributions presented in the study are included in the article/Supplementary Material, further inquiries can be directed to the corresponding author/s.

## Author Contributions

All authors listed have made a substantial, direct, and intellectual contribution to the work, and approved it for publication.

## Conflict of Interest

The authors declare that the research was conducted in the absence of any commercial or financial relationships that could be construed as a potential conflict of interest.

## Publisher’s Note

All claims expressed in this article are solely those of the authors and do not necessarily represent those of their affiliated organizations, or those of the publisher, the editors and the reviewers. Any product that may be evaluated in this article, or claim that may be made by its manufacturer, is not guaranteed or endorsed by the publisher.
